# The lived experience of weight loss maintenance in young people

**DOI:** 10.1111/hex.13955

**Published:** 2023-12-28

**Authors:** Nicola Sides, Andy Pringle, Lisa Newson

**Affiliations:** ^1^ Ofcom London UK; ^2^ Previously Carnegie School of Sport Leeds Beckett University Leeds UK; ^3^ School of Human Sciences, Centre for Clinical Exercise and Rehabilitation, College of Science and Engineering University of Derby Derby UK; ^4^ School of Psychology, Faculty of Health Liverpool John Moores University Liverpool UK

**Keywords:** interpretative phenomenological analysis, intervention, obesity, qualitative, weight maintenance, young people

## Abstract

**Introduction:**

There continues to be an imbalance of research into weight loss and weight loss maintenance (WLM), with a particular lack of research into WLM in young people under 18 years. Failure to coherently understand WLM in young people may be a potential contributor to the underdeveloped guidance surrounding long‐term support. Furthermore, no research has investigated young people's preferences around WLM support following the attendance of a residential intensive weight loss intervention from a qualitative perspective. This study explored the influences of WLM in young people following a residential intensive weight loss intervention, considered how interventions could be improved and sought to develop recommendations for stakeholders responsible for designing WLM interventions.

**Methods:**

The context in which this research is framed was taken from a residential Intensive Weight Loss Intervention for young people aged 8–17 years in England. Six semi‐structured interviews were carried out to understand the lived experience of WLM, including barriers and enablers influencing WLM, adopting an interpretative phenomenological analysis design.

**Findings:**

Three superordinate themes were developed to explain the barriers and enablers to WLM; (1) Behavioural control and the psychosocial skills to self‐regulate WLM; (2) Delivering effective social support; and (3) Conflicting priorities and environmental triggers.

**Conclusion:**

The findings of this research mirror that of other studies of WLM in young people, with the majority of young people struggling to maintain weight loss. However, by exploring the experience of WLM in young people through qualitative means, it was possible to understand the specific motivators and barriers influencing WLM behaviours in this context, providing recommendations to support WLM.

**Patient or Public Contribution:**

The interview guide was developed in consultation with a young person from the intervention, and through discussions with the intervention stakeholders (delivery staff and management staff). The interview guide included topics such as knowledge and skills; experience of weight loss; reflections on weight maintenance, and experiences of daily life postintervention. We piloted the interview schedule with one young person who had consented to take part in the research. This first interview was used to check for understanding of questions and to assess the flow of the interview.

## 1 INTRODUCTION

Addressing overweight and obesity in young people under 18 is a pressing public health concern, with one‐third of children aged 2–15 in England classified as overweight or obese.^1^ The associated comorbidities persist into adulthood,^2^, ^3^, ^4^ emphasising the need for sustainable weight loss interventions.

Multicomponent weight loss interventions are recommended for overweight and obese young people with complex needs and comorbidities.^5^ National Institute for Health and Care Excellence (NICE) recommends interventions should be delivered in various formats including group support, individual and weekly programmes. Alternatively, for some children and young people with obesity, a more intense intervention, such as a residential intervention, may be necessary.^6^ Residential interventions claim to educate and upskill young people in a focused environment, whereby postintervention, the young people continue to implement the new skills and knowledge at home and in their daily lives.^7^ The outcomes of such intense interventions show promise,^8^, ^9^ presenting more significant weight loss benefits to young people than those attending weekly community lifestyle interventions.^10^ Clinical outcomes show various health improvements such as reductions in weight, body mass index (BMI) and BMI standard deviation scores (BMI SDS).^8^, ^11^, ^12^ However, there lies one overriding and essential issue: the maintenance of weight loss.^13^, ^14^, ^15^, ^16^

Weight loss maintenance (WLM) poses a significant challenge, with only about 20% of adults successfully sustaining their weight loss,^17^, ^18^ a difficulty also extending to young people.^19^, ^20^ A systematic review Kelly and Kirschenbaum^20^ evaluated in‐patient settings for childhood and adolescent obesity, revealing mixed outcomes among 22 studies. Eleven studies follow‐up participants at periods ranging from 4 months to 3.6 years. Weight gain was reported in seven studies, significantly so in three. The other four studies reported continued weight losses. These findings further argue WLM in young people is an area requiring further research, in particular influences affecting WLM. Another review^19^ evaluated 11 studies and concluded that maintenance interventions benefited WLM but that there is limited quality data to recommend one intervention over another. This underscores the need for ongoing research to guide intervention design. Of the studies included in both systematic reviews,^19^, ^20^ no studies of WLM were carried out in the United Kingdom.

Despite limited research on WLM in young people, there are only two qualitative studies in the literature on residential intensive weight loss interventions for young individuals.^21^, ^22^ Neither of these studies^21^, ^22^ explored how the young people would like to be supported following the residential programme, nor the experience of a WLM intervention. Qualitative studies provide valuable insights in less‐explored areas, enhancing understanding. One study^23^ recommends that evaluations not only assess what works, but also why, providing essential feedback for intervention developments.

While several studies have evaluated children's experiences of attending weight management programmes,^21^, ^22^ none, to the author's knowledge, specifically explore young people's experiences with WLM interventions in the United Kingdom. From the parent's perspective, studies suggest a lack of support post ‘treatment’ period.^24^ Barriers identified the extended family as undermining efforts,^24^ and monetary and time costs affecting engagement in weight loss and ongoing maintenance.^25^

Current UK guidelines for weight management interventions recommend follow‐on support for a minimum of 12 months.^26^ However, details on how providers deliver this support are limited, lacking guidance on content, delivery or frequency. However, an umbrella review (a review of reviews) reports that existing evidence typically only includes a research or clinical follow‐up, focusing on anthropometric measurements without clinical support sessions to facilitate WLM.^27^

The support mechanisms to engage in WLM remain under‐researched, necessitating evidence to understand young people's needs, motives, expectations and barriers to achieving WLM.^28^, ^29^ Additionally, understanding stakeholders, including young people's preferences for maintenance delivery, is an essential requirement of intervention mapping. The ultimate impact of a programme depends on its effectiveness, reach and ongoing implementation within the target population.^30^

## 2 AIM AND OBJECTIVES

This study explored the experiences of WLM in young people following a residential intensive weight loss intervention, considering how interventions could be improved to develop recommendations for stakeholders responsible for designing interventions to support WLM.

## 3 METHODOLOGY

### 3.1 Context: Intervention

This research has recruited young people (aged 8–17) from a residential intensive weight loss intervention in England. Eligibility to attend the intervention was for young people classified as overweight or obesity (on a BMI SDS at, or above, the 85th centile in line with the centiles set out in UK90).^31^ Referrals to the intervention were made by a health professional (e.g., school nurse, general practitioner [GP] or dietician) or through self‐referral.

The residential intervention in Northern England lasted up to 6 weeks, with residential stays varying from 1 to 6 weeks, during the British summer school holidays (July–August). The programme followed a lifestyle intervention (Table 1),^35^ combining physical activity (PA), dietary restriction and lifestyle education.

**Table 1:** Intervention content (MoreLife, https://www.more-life.co.uk, summers of 2012–2014).

*Physical activity* (PA). The role of PA in weight loss has been well documented^32^ with components relating to self‐efficacy increasing long‐term behaviour change.^33^ With this, the PA sessions aimed to be fun, encouraging young people to see PA as a positive aspect of life and develop skills to be physically active.
*Dietary restriction*. Young people received three daily meals and an afternoon fruit snack. Each young person's dietary intake was tailored on a calorie allowance calculated from their estimated basal metabolic rate.^34^ Daily energy intake was designed to modestly reduce body mass through energy intake expenditure imbalance, while providing enough calories to maintain growth and sustain energy for the requirements of the Intervention.^7^
*Lifestyle education*. The purpose of the lifestyle sessions was to educate young people about healthy living and prepare young people for a healthy lifestyle following the intervention programme. Content included nutritional and behavioural education through, for example, goal setting, self‐monitoring, and problem‐solving.

After leaving the intervention, participants transitioned to the 12‐month follow‐on programme aligning with NICE guidance for maintenance.^26^ Support was provided to the young people through telephone calls, text messages, digital support via the organisation's own website with members login, social media and in‐person support (NHS commissioned community interventions and home visits available for UK residents for research purposes to obtain anthropometric measurements) (Table 2). Support was designed to be knowledge‐based featuring reminders of recipes, lifestyle education and workouts. It also included prompts to enact self‐regulation behaviours, like self‐monitoring of weight, goal setting, problem‐solving, all aimed at motivating and sustaining lifestyle behaviours.

**Table 2:** Details of the weight loss maintenance intervention timeline (MoreLife, https://www.more-life.co.uk, summers of 2013–2014).

Weight loss maintenance intervention	Support available
1–12 Weeks following departure from the intervention.	Community weight loss programme (1x week). Telephone call (1x week). Text messages (3x week). Web support (24/7 support). Social media (24/7 support). Home visit (one visit: 3 months postintervention).
12 Weeks–12 months following departure from the intervention.	Web support (24/7 support). Social media (24/7 support). Home support visit (two visits: 6 and 12 months postintervention)

The follow‐on programme was designed to provide a gradual decrease in support, optimising self‐efficacy and encouraging self‐regulation of their weight management, a key determinant of long‐term success.^36^, ^37^

### 3.2 Research design

This study adopted an interpretative phenomenological analysis (IPA) design allowing a detailed examination of the human lived experience,^38^ to explore the experiences, perceptions and motivations for WLM in young people. IPA is described by Smith et al.,^38^ as ‘an approach to qualitative, experiential and psychological research which has been informed by concepts and debates from three key principles of philosophy of knowledge: phenomenology, hermeneutics and ideography’ (p. 11). All three principles were upheld in the current study with the researcher taking an insider role,^39^ immersed within the research to fully understand the intervention, and the experiences of the young people.^40^ IPA is unique to other qualitative methodologies in that it is concerned with the particular rather than the universal in an experience. Within this research, interviews were selected to access the young people's ‘perceptions, meaning, definitions of situations and constructions of reality’.^41^

Given the lack of research into young people's experience of WLM from the perspective of the young people, IPA was deemed most appropriate to allow for a detailed and in‐depth analysis.^42^ Within IPA, ‘fewer participants examined at a greater depth is always preferable to a broader, shallow and simply descriptive analysis of many individuals’.^43^

### 3.3 Participants and recruitment

Semi‐structured interviews were carried out to understand the lived experience of WLM, including barriers and enablers with the aim to provide recommendations for how best to support WLM in young people.

Invitations to interview were advertised 11 months postintervention using Facebook and text messages, delivered as part of the follow‐on support programme provided to the young people following the residential intervention. Seven young people responded, with one individual dropping out. A sample of six young people (*n* = 3 boys, *n* = 3 girls; mean age: 13.17 ± 1.72; range: 11–15 years, mean stay: 3.83 ± 1.84 weeks, range: 1–5 weeks) agreed to take part in interviews to discuss their experience of WLM and preferences for follow‐on care. Of this sample, three were deemed to have maintained weight loss 12 months postintervention, indicated by a BMI SDS below their preintensive weight loss intervention measurements. Of those, two young people continued to lose weight and one gained weight but remained below preintervention measurements. The remaining three gained weight above their preintervention measurements. This was representative of the typical WLM achieved across the intervention attendees.^44^ In line with IPA, this small sample was deemed to be homogeneous and representative of the young people attending the intervention, examining these participants' experiences in‐depth.^45^

### 3.4 Measures and procedures

Twelve months postintensive weight loss intervention, semi‐structured interviews were conducted. Participants were offered flexible appointments to participate. Three interviews were conducted in person, and three via telephone.

The interview guide (see Supporting Information SA) was developed in consultation with a young person from the intervention and intervention stakeholders (intervention staff), referencing relevant WLM and behaviour change research literature. The interview guide included topics such as knowledge and skills; experience of weight loss; reflections on maintenance, experiences of daily life postintervention. The interview guide underwent a pilot with the first participant consenting and helped to check question understanding and flow. No significant changes were made following the pilot, and this interview was included in the final sample and analysis.

### 3.5 Ethical considerations

Institutional ethical approval was obtained from the Research Ethics Committee at Leeds Beckett University. Participants were reminded of the research nature focusing on their postintervention experiences. Both young individuals and their parents had time to decide participation, address questions or concerns, with consent secured from participants and parents.

Before the interviews, participants were reminded of ethical consent and the right to withdraw. The interviewer maintained neutrality, empathy and nonjudgmental, employing active listening^46^ to promote free expression. Postinterview, debriefing addressed any questions or comments and participants were thanked. Interviews averaged 34 min (range: 16–46 min). The lead researcher kept a reflective journal to note initial evaluations and assumptions postinterviews.

## 4 RESEARCH TEAM

The lead researcher (N. S., PhD student and Trainee Health Psychologist at the time) conducted the interviews, and took the lead role in the analysis, was also a staff member at the intervention. This research was conducted as part of a wider PhD whereby the lead author engaged in a participatory action research methodology, immersing themselves in the programme. This allowed the researcher to learn more about the intervention and follow‐on programme than one who would solely learn from the interviews. As part of methodology process and built into researcher reflection, care was taken to ensure participants did not feel coerced to participate. Participants were reminded of the researcher's role throughout, and that participation was voluntary. Young people interacted, and built a rapport, with the lead researcher throughout the intervention. We believe, this prior relationship acted as a strength in this qualitative research offering comfort and trust for the interview, encouraging the young person to quickly open up and offer rich and more honest insight into their experiences, which they may not have offered if engaging with a new unknown researcher.^47^ The study was codesigned and supervised by a professor of PA and health intervention (A. P.) and a reader in applied health psychology and registered practitioner psychologist (L. N.).

### 4.1 Analytical procedure and quality

Interviews were digitally recorded, transcribed verbatim, and the lead researcher removed identifiable information. The analytical procedure followed that recommended within IPA procedures^38^ (see Table 3). Throughout this process to ensure quality, these fluid stages of analysis were discussed across the research team, and in addition the researcher reflections developed throughout this research, were integrated into the analytical process, and brought for discussion with the research team to aid analytical triangulation.^48^ IPA applies a double hermeneutic perspective, enabling the researcher to interpret participant experiences. The lead researcher acknowledged personal and professional experiences throughout the intervention and follow‐on care. As IPA follows an idiographic approach,^49^ each case was considered singularly for themes before moving to another. The transcripts were checked to confirm that the themes reflected the content of the interview. Themes were prioritised considering both prevalence in the data sets, richness and importance to the research question. Recurrent themes were then explored to enhance validity^38^ ^,p.107^ and presented within a table (Table 3, see Supporting Information files e.g., analytical process). A map of themes and superordinate themes was created once all interviews had been analysed.

**Table 3:** Stages of analysis.^38^

Stage	Description
1. Reading and familiarisation	Each interview transcript was repeatedly read and listened to while initial, unstructured notes were made. Recollections of interviews and observations about transcripts were documented.
2. Initial noting	Exploratory descriptive, linguistic and conceptual coding was initiated throughout each transcript. Notes, including reference to the postinterview reflective commentary summarising participant experiences, were added to each transcript (1 and 2 iterative process).
3. Developing themes	Notes were reviewed and theme titles, along with quotes, were developed, to support analytical commentary.
4. Searching for connections across themes	Subsumption, abstraction, numeration, polarisation and function methods were used to consider themes and cluster and merge themes together.
5. Repeat steps 1–4	Moving to next case, repeat steps 1–4. Step 4 across all data.
6. Patterns within and across themes	Patterns in master themes were identified across cases to identify superordinate and subordinate themes.

## 5 FINDINGS AND DISCUSSION

We have merged the results and discussion in terms of consideration of the analytical findings together with the interpretation of the current evidence base relevant to the themes presented. This approach is with consideration to the flexible nature of IPA^38^ and encompasses the theoretical and conceptual contexts within each theme, as opposed to repeating findings within the discussion.

Of the six participants, only one continued to lose weight following the intensive weight loss intervention without any periods of weight gain, while the other participants struggled to maintain their weight loss at times. The participants described WLM as difficult and attributed their success and struggle to maintain their weight loss to several barriers and enablers to WLM. These barriers and enablers are presented in three superordinate themes; (1) Behavioural control and the psychosocial skills to self‐regulate WLM; (2) Delivering effective social support; (3) Conflicting priorities and environmental triggers.

Theme 1 *Behavioural control and the psychosocial skills to self‐regulate WLM*

When returning to the home environment, all participants referred to high intrinsic motivation enabling WLM, resulting from increased confidence, self‐efficacy, and self‐esteem during the intervention. High levels of self‐esteem and self‐efficacy were sustained through performance accomplishments and verbal encouragement, facilitating positive emotional states.

I found it a lot easier at first because everything was quite fresh in my mind and it was like wanting to put everything into place … I was into my football so I wanted to always be doing that. (Participant 6, female)

I went back to school and people were like talking [about me]. And I just used to have a cheeky little grin walking by, and it was just everyone saying well done … I got a lot of feedback from that from people saying I was inspiring what you've done. (Participant 3, male)

Participant 6, here expresses a positive emotional relationship with the new lifestyle skills and behaviours, she developed during the intervention. Similarly, Participant 3 presents positive emotional undertones resulting from compliments from peers. These positive experiences show a perceived connection between high self‐esteem and self‐efficacy with maintenance of WLM behaviours.

Participants reported an initial lapse in behaviour coinciding with the discontinuation of regular communication (follow‐on phone calls) by the intervention staff, which ended 12 weeks after the residential intervention. Despite the tailored goal to equip young people with psychosocial skills for independent WLM in challenging situations, three participants lost their sense of purpose.

Once the follow‐on care ended it's like, that's it. It's just a memory … some lessons you've learned … but I wasn't really sticking to them… Because the follow‐on care had stopped, it was just like I didn't, I never really had a purpose to carry on with stuff like that. (Participant 3, male)

There was a lot of support to begin with and then it gradually just went down and down. Especially when [intervention staff] stopped the [calls], it just went bang. It hit the ground. (Participant 1, male)

Participant 3 reflects a sense of closure and loss after the follow‐on care ended describing the intervention as a mere memory with lessons learned but struggles to adhere to them without ongoing support. The cessation of follow‐on care leaves the participant feeling purposeless, lonely and disengaged from the goal of maintaining weight loss. Participant 1 highlights a decline in support over time, perceiving a sudden drop in assistance. The metaphorical description of the support hitting the ground conveys a dramatic and negative impact on the participant's experience. This suggests a critical dependence on external support, and the abrupt discontinuation has a profound effect on the participant's well‐being. Both quotes indicate the significance of continuous support in maintaining positive outcomes postintervention. The participants express feelings of disconnection and a lack of purpose when this support diminishes, emphasising the need for sustained assistance to facilitate lasting behavioural changes.

A perceived lack of behavioural control^50^ provides one explanation for this change in attitude. This theory suggests that the young people do not fully endorse their behaviours, instead identifying the perceived source of initiation and regulation of a behaviour outside one's self^51^ that is, through the intervention staff whom the young people perceive as an authoritative figure whom they were maintaining weight loss for. This relationship is not surprising given the power divide between children and adults in wider society whereby children frequently behave in response to the requests and directions of adults (e.g., parents and teachers). It is therefore likely young people have little or no experience of having to control and self‐regulate their behaviour and are therefore ill‐prepared for long‐term behaviour change. Perceived control is of importance as this has been shown to be a stronger predictor of WLM success than motivation.^52^

Participants sought extrinsic motivation from the authoritative relationship with intervention staff during the maintenance period. They actively sought positive reinforcement for weight maintenance but avoided seeking support when not adhering to the programme. Among those regaining weight, all acknowledged hesitance to contact the intervention staff for assistance during this period.

It put me in that that mind frame that I was letting down [the staff]. Sometimes I couldn't message him because obviously, I'd gained weight … I was more scared to tell him that I felt I'd let him down. (Participant 3, male)

Participant 3 encapsulates a complex interplay of emotions within the context of the participant's relationship with the staff. The participant describes a heightened sense of accountability and a perceived obligation not to disappoint the staff, indicating a strong emotional connection. The fear of conveying weight gain prevented open communication, highlighting a vulnerability and reluctance to admit perceived failure. The mention of being ‘in that mind frame’ suggests a psychological impact, possibly reflecting a heightened emotional state associated with the participant's self‐perception and the perceived expectations of the staff. The participant's hesitancy to reach out for support stems from a fear of judgement and the potential acknowledgement of falling short of expectations. The relationship with the staff extends beyond a mere functional support system; it carries emotional weight, influencing the participant's self‐image and communication patterns. The participant's narrative emphasises the need for a supportive and understanding environment that encourages open dialogue, mitigating the fear of judgement and fostering a collaborative approach to addressing challenges.

Participants assumed they were the only one struggling with WLM, preventing a social support system and shared identity.

I don't want to see [my intervention friends] until I've lost the weight again. (Participant 3, male)

The choice of words, particularly ‘don't want to see’, suggests an interpretative link between the participant's identity and social acceptance, implying a certain level of discomfort or reluctance. There may be emotional nuances associated with the participant's current state of appearance that influences their desire to avoid social interactions with this specific group. Whilst developing a shared social identity has been found to increase engagement and health outcomes in weight loss,^53^ this concept of the participant avoiding peers in response to weight gain aligns with the growing body of evidence surrounding weight‐based social identity threat where an individual perceives they will, or have been, devalued or discriminated against because of their weight^54^, ^55^ motivating avoidant coping strategies, contributing to weight gain. Given the research showing weight gain following weight management interventions and the influences affecting motivation discussed within this theme, more attention is required to protect self‐esteem, self‐efficacy and self‐regulatory behaviours if young people start to gain weight.

Theme 1 explores participants' struggles with self‐regulation in WLM. Challenges surfaced when intervention staff‐initiated contact ceased, underscoring the need for ongoing social support. This is crucial, especially for individuals, like children, who often respond to authoritative figures such as parents or teachers. Without the psychosocial skill to transfer learning into everyday life, WLM is unlikely, irrespective of the intervention content. It is recommended to educate young people on behavioural control and self‐regulation for weight maintenance.^36^, ^37^, ^56^, ^57^, ^58^

WLM requires significant psychological tools to maintain motivation, and self‐regulate behaviours, made more difficult given the stigma attached to obesity. This is something most adults find difficult and we consider this to be even challenging for younger people, with little to no experience of autonomy, given the role parents and teachers play in directing their actions in life. Commissioners must recognise the cognitive burden this puts on young people and enable weight loss interventions to continue to deliver ongoing support. Emphasis on psychosocial skills (e.g., self‐regulation) and support systems to guide and support the young person is crucial for effective WLM.

Theme 2 *Delivering effective social support*

Social support is a known facilitator to WLM,^59^, ^60^, ^61^ reinforced through all participants describing positive experiences from both staff and peers at the intervention and its facilitating role to WLM. The absence of social support posed challenges to weight maintenance. Interestingly, participants avoided social support when they had gained weight. To reduce the risk of avoidance behaviours, the participants cited preferred features of ongoing support affecting their motivation to engage in the follow‐on support. A continuation of care was identified as a favoured feature of WLM support, having built a rapport while attending the intervention.

You know, seeing the faces again … then I get weighed by someone that you know and trust instead of some randomer. It was good, yeah. (Participant 1, male)

I think it was just nice to hear like a friendly voice. It someone you know, that you've spent the summer with, you've got to know them they've got to know you. (Participant 3, male)

The participants provide insights into the significance of familiarity and interpersonal connection within the context of their weight loss journey, emphasising the importance of being weighed by ‘someone that you know and trust’. This reflects a sense of security and comfort associated with familiarity in a potentially vulnerable situation. There may be a belief that a familiar face contributes to a more trustworthy and supportive intervention, contributing to an overall more positive experience. Continuity of care has been linked to long‐term obesity treatment, reinforcing the importance of continued support as the most highly rated component with continued support predicting weight loss success.^60^ In summary, both participants highlighted the importance of familiar faces, trust and positive social interactions in their experiences with the intervention. The phenomenological aspects capture the immediate sensory and emotional dimensions, while the interpretative aspects delve into the participants' beliefs about the significance of familiarity and positive interpersonal connections in the context of the intervention.

Despite appreciating the continued telephone support, one participant found it monotonous and would have preferred a less structured conversation.

Yeah, it's the same questions … it's just like how have you been feeling and that and then it's just the same questions every time. (Participant 1, male)

The participant's mention of ‘the same questions every time’ suggests an interpretative aspect related to routine and predictability. There may be a perception that the intervention follows a standardised format, which is inconsistent with changes to their motivation, and conflicting priorities (discussed in Theme 3) potentially affecting their engagement with the process. A semi‐structured interview guide, designed to aid weight management practitioners through the ongoing support, may have inadvertently acted as a barrier to the young people expressing concerns when not asked directly. This may have further reinforced the control the intervention staff had over the programme, rather than allowing the young person to take the lead in their WLM journey.

Throughout the interviews, the presence of a supportive figure whether ‘seeing the faces again’ or ‘to hear a friendly voice’ was consistently reported above that of knowledge‐based advice, emphasising the need for positive reinforcement. Further research is required to understand the transition of this supportive role to prevent a significant shift in WLM behaviours. In summary, the role of the intervention staff and their influence on self‐efficacy and continued WLM behaviours was evident in all interviews, often at length, compared to minimal detail of proposed knowledge‐based content, evidencing the value based on motivation rather than education in WLM support. Future guidance for WLM should address this for effective ongoing support.

Theme 3 *Conflicting priorities and environmental triggers*

Weight regain is not unexpected when returning home due to social and environmental cues promoting unhealthy behaviours.^62^ Despite initial success in maintaining weight loss in the home environment, fuelled by self‐efficacy and social support, these reduced in time suggesting these were not a learned priority, indicating a requirement for ongoing education and support.

Yeah, quite prepared to begin with and then obviously over time … you begin to forget it. (Participant 1, male)

Instead, participants referred to conflicting priorities and environmental triggers interfering with WLM behaviours, notably education and seasonal activities.

During the exam season, I didn't [play football]. (Participant 5, female)

When it comes to revising and stuff, I realised that I wasn't eating my [healthy] meals. (Participant 6, female)

And then it's like, oooo, Halloween … then it gets to Christmas, and you're like ah, and then you just overeat, and it doesn't go off, and then it's my birthday nine days afterwards (laughs). (Participant 1, male)

The participants' choice, not to play football during the exam season, or to neglect healthier eating behaviours, suggests an interpretative aspect related to prioritisation. There may be a perception that academic demands took precedence over recreational (lifestyle/and, therefore weight management) activities during this period. The participants' narrative may relate to cyclical eating behaviour tied to specific occasions. There may be a belief that these events trigger overeating, forming a recurring pattern which they struggled to get out of.

The laughter within the final quote indicates a sense of awkwardness, in that the participant knew they were making excuses and had not prioritised their weight loss. Another participant reflected on their experience:

Obviously then I thought I was really busy, and then I look at it now, and I think Jesus I had absolutely nothing to do. All my priorities were in the wrong place. (Participant 3, male)

Here, Participant 3, reflects on a shift in perception, moving from the belief that he was ‘really busy’ to a current realisation that he had ‘absolutely nothing to do’. This suggests a subjective and evolving experience. The participant's current perspective, expressed as ‘I look at it now’, links to his learning and growth. There is recognition of personal development and a shift in understanding over time, to understand his own lifestyle/needs.

Although participants reflected on their WLM and prioritisation of weight maintenance behaviours, they lacked coping mechanisms to deal with triggers. Conflicting priorities revealed dichotomous thinking about WLM; something you do, or not, rather than a gradual behavioural change. Such polarised thinking may limit flexibility, making WLM more difficult.^63^, ^64^

Research acknowledges seasonal weight gain in children, particularly during Christmas and school holidays, impacting healthy behaviours due to increased social gatherings, energy‐dense foods, a more carefree lifestyle and less PA.^65^, ^66^ The intensive intervention aimed to empower individuals to manage such seasonal challenges. However, despite recognising holidays as a challenging time, and incorporating problem‐solving lessons into the intervention, participants were not equipped to implement these strategies in real‐world situations.

This theme supports the need for national guidance for ongoing psychosocial support, especially during challenging periods for WLM. Specific support should target self‐regulation techniques such as goal‐setting, self‐monitoring, problem‐solving and making plans to avoid and/or respond to lapses.^64^

## 6 DISCUSSION

This study adopted an IPA research design to explore the lived experience of WLM in six young people following a residential intensive weight loss intervention. Although previous studies of WLM in young people have taken the perspective of the parents,^24^, ^25^ others Robertson et al.,^28^ Nobles et al.,^29^ highlight the value of young people in the development of their care and should be involved in the evaluation process. This is the first study to use qualitative methodologies to understand WLM from the perspective of the young person experiencing it.

This study unearths the complex relationship young people have with WLM, whereby they are required to take control of, and self‐regulate, WLM in a culture where their behaviours are typically is response to the direction of an authoritative adult. This highlights the need to explicitly address psychosocial skills to equip young people with the tools necessary to maintain WLM behaviours. As highlighted in a recent review article,^67^ interventions are currently failing to fully address the psychological needs of children and young people, changes to practice are required.

It is understood that returning to the home environment for participants is difficult,^11^, ^62^, ^68^ and relapse in behaviours and weight regain is typical following obesity treatment in both adults^17^, ^18^, ^60^, ^69^ and young people.^20^, ^70^ This study revealed similar findings, most of the participants struggling to maintain their weight loss at some time throughout the following 12‐month period. Young people identified challenges in overcoming social and environmental triggers (e.g., winter and seasonal celebrations) and conflicting priorities (e.g., schoolwork and exams) as well as maintaining motivation in the absence of positive feedback and reinforcement provided by the intervention staff, in the form of social and psychological support.

These findings highlighted the value the young people placed on an authoritative figure as a guiding force to maintain weight loss, and the requirement to prioritise psychosocial skills to self‐regulate behaviours in the face of social and environmental triggers, yet in the absence of professional support.

In summary, the role of a supportive, authoritative figure, and the influence on self‐efficacy and continued WLM behaviours was addressed in all interviews, often at length, compared to minimal detail of proposed content, suggesting the support of the intervention staff to be an important consideration in WLM support, more so than the educational content of a WLM intervention.

This research highlighted the scarcity of explicit guidance for maintenance following child weight management services. To address this, a number of recommendations (Table 4) have been proposed, which should be considered for the development of WLM interventions, and national guidance for young people.

**Table 4:** Recommendations for national guidance and commissioning services.

1. Explicit guidance is needed for long‐term support detailing what WLM is in young people (this should consider: delivery mechanism, content, duration, frequency).
2. Commissioners need to recognise the psychological burden on young people to maintain long‐term behaviour change, with specific support dedicated to psychological skills to self‐regulate behaviour and maintain motivation, for example, goal‐setting, self‐monitoring, problems solving and making plans.
3. Additional support should be resourced during expected periods of difficulty (e.g., Christmas, winter season).
4. A continuity of care is recommended whereby professionals have built a rapport with the young person and understand their individual needs and circumstances.
5. More research is required to understand young people's relationships with authoritative figures (e.g., parents/caregivers and healthcare professionals) and how best they can support young people maintain weight loss.
6. Support should be flexible and tailored to the young person's needs. This may include frequency and length of support.
7. To equip an authoritative figure (e.g., caregiver) with the necessary skills and education to support young people's WLM in the absence of intensive weight loss intervention staff following a weight loss intervention.
8. Intensive weight loss intervention to include psychological support, including problem‐solving, behavioural control, the responsibility of behaviour and self‐regulation to support WLM.

Abbreviation: WLM, weight loss maintenance.

### 6.1 Recommendations for future research

Future research should consider the young people's perceived control over their WLM behaviours to enable self‐regulation and maintain intrinsic motivation. Further research surrounding authoritative figures and how they can support young people's WLM is also warranted. Finally, further investigation to explore the transition periods from the intervention setting to their home environment, and from dedicated professional support to self‐regulation is required.

### 6.2 Strengths and limitations of the research design

This qualitative research delves into the experiences of six young people who have engaged in WLM. Given the scarcity of young people's voices in WLM research, this study provides a unique and essential viewpoint.^67^ Using IPA, the research explored an under‐researched and sensitive topic of WLM in young people. Following IPA methodology,^38^ a small homogenous sample of young people shared lived experience of having completed a specific weight loss intervention, and the subsequent 12‐month postintervention WLM period. The researcher's immersion into the intervention allowed for a heuristic approach, crucial in IPA.^38^ Quality was ensured through utilising researcher reflection, triangulation and the use of verbatim quotes used as evidence to enhance the validity of the superordinate themes and represent the voices of participants. It is important to note that these experiences may not be universally representative and should be cautiously applied to other weight management interventions across the United Kingdom. Nevertheless, these rich insights highlight areas for further exploration and service improvement to enhance the lived experiences of young people undergoing weight loss or maintenance.

## 7 CONCLUSION

The findings of this research mirror that of other studies of WLM in young people, with the majority of young people struggling to maintain weight loss. However, by exploring the experience of WLM in young people through qualitative means, it was possible to understand their lived experiences, and to consider the specific motivators and barriers influencing WLM behaviours in this context. In particular, the overriding theme of motivation discussed by all participants and its driving role in maintenance behaviours, compared to minimal reference to education or tools and resources to maintain weight loss, suggesting psychosocial processes (e.g., self‐esteem, self‐efficacy) to be the most important consideration in WLM support in this cohort. Furthermore, because young people remain heavily dependent on authoritative others (e.g., parents, teachers, and in the case of this research, intervention staff), their role and influence on motivation and WLM in young people should be explored further and recognised within WLM intervention design.

This study serves to contribute to the limited evidence base concerning WLM in young people, with particular attention to the considerable psychological challenges young people face when maintaining weight loss. This highlights a significant gap in the national guidance for WLM and provides useful insights for stakeholders which can influence future intervention design for childhood WLM.

## AUTHOR CONTRIBUTIONS

**Nicola Sides**: Conceptualisation; methodology; investigation; writing—original draft; writing—review and editing; data curation; validation; formal analysis; project administration. **Andy Pringle**: Methodology; conceptualisation; writing—original draft; writing—review and editing; supervision; formal analysis; validation. **Lisa Newson**: Supervision; writing—review and editing; writing—original draft; formal analysis.

## CONFLICT OF INTEREST STATEMENT

The authors declare no conflicts of interest.

## ETHICS STATEMENT

Ethical approval was granted by Carnegie Faculty of Sport and Education, Leeds Beckett University. To promote transparency in context, the findings section of this manuscript presents verbatim quotes from a range of the participants to act as evidence to support the analytical commentary. In recognition of legal and ethical processes, participants of this study did not agree that their transcripts were fully shared publicly, so supporting data beyond the sample quotation extracts is not available.

## Supporting information

Supporting information.

Supporting information.

## Data Availability

Raw data have been included as evidence via extracted quotes from verbatim transcripts as samples of evidence. Full transcript release has not received ethical approval or participant consent. For further study details, please contact the corresponding authors. The authors confirm that the data supporting the findings of this study are available within the article.
