# Supplementary material for: The lived experience of weight loss maintenance in young people
Source: Health Expect. 2023 Dec 28;27(1):e13955. doi: 10.1111/hex.13955 (PMC10768871; doi:10.1111/hex.13955)
Supplement: Supplementary file 1 — Supporting information. [file HEX-27-e13955-s002.docx]

**Step 2 & 3: Initial noting and Development of themes**

| So how was life after camp? Thinking about when you first left camp, did you think that…?   - Easy (pause) to begin with, and then it gets harder   When did it get harder?   - Christmas. and then it's like, oooo, Halloween is not as bad because you can do other things other than trick-or-treating and then it gets to Christmas and you're like ah and then you just overeat and it doesn't go off, and then it's my birthday nine days afterwards (laughs)   And it's winter as well   - yeah so we didn't really offer much help   Did it get any easier like after Christmas when it started to get to summer   - yeah in the summer it did and a lot of it came off and then it came straight back on, and it hasn't really come off this year.   So with like you said when you got back it's pretty easy what was easy at first?   - The portion sizes, and the exercise on that because I knew what I was doing and I knew that because I knew I could do it, but then it got later on I was forgetting things so I think this is just to reinforce things so I don't forget it because I lost my folder last time and then it was like yeah so I'm gonna put this on my bookshelf this time   Do you think that if you'd had your Handbook, you knew where your handbook was, it would have been…   - Yeah I think it would have been different [if I had my handbook], it would have helped. Maybe not loads but some helped a lot.   Yeah, and what did you find the hardest?   - Probably my confidence going down, seeing as when I stop in the weight on my confidence went back down, and then I don't now it's just confidence levels going down again. Hardest thing about it.   What do you think, was there anything on camp that helped in particular with like life after camp to make you more prepared?   - The lifestyle sessions and the think good, feel good. That was really good. Giving us the right emotions and that because like when we did the [group self-esteem session] and we put nice things and like with a group but you know like was supportive and safe and had your back and things like that. It was a nice environment to be in.   Was there anything else you think more life could have done to help you feel more prepared for going home?   - I don't know because you'd already prepared us enough, as you could without you know giving us book after book after book and overwhelming us with information. You've given us more than just the basics. Maybe like a complex version of it just to get our heads round and then give us the equipment and information that we need to go.   Yeah. Did you feel quite prepared?   - Yeah quite prepared to begin with and then over time you lose the, you begin to forget and   So forgetting. How was motivation   - it was good to begin with   Did that change over…?   - Yeah, I had no motivation for the past year. I struggled a bit.   Why do you think you struggled with motivation?   - I don't know, it's just my weight went back up, like feeling down most of the time, it just got really hard and then I just gave up.   ……….  What about support after camp, did you feel supported? When I say this I mean [intervention staff], I mean by family, friends   - Yeah, yeah it was, no there was a lot of support to begin with and then it was like it gradually just went down and down and down. Especially when [intervention staff] stopped the support, it just went bang. It hit the ground and then it just stopped. I mean [my guardian has] always been supportive and he's always been there for me and I mean the past few months he's been saying, we need to get something done you know your motivation, I think you can get something done. You know it’s always been quite kick it up your arse and he's been good about that. And my grandma's been the same. I haven't been listening to them.   Why are you not listening to them?   - I don't know. Getting back into bad habits I guess   What sort of bad habits are you getting back into?   - Being lazy. Sleeping in until like 11 o'clock. Especially over summer I haven't been getting up until like 10, 11. Then kind of think to yourself yeah I slept into 11 great. I've kind of wasted today. I mean obviously hate you're up at what say 7:30, you've done loads.   Do you feel any better for actually doing something with your day?   - Yeah I mean I lost two KG something like that and just like coming off eventually, I mean the weight I am, I struggle to keep it off and it's not a struggle to get it back on. Is easy to put it back on.   Yeah it's easier to put on and take back off. Why do you think you find it much easier to gain rather than lose?   - Because like there's Temptations and that because I don't have any energy most the days and just like. Whenever I'm at school at like break and lunch we play football and things like that and we are always running around. But as soon as you get home and your mates are like on Xbox or whatever I’m just like ‘are you going out?’ ‘No’. Right I’ll just play Xbox with you so it's easy   So it's easy to just stay in and play Xbox?   - Yeah, rather than being by myself or something. I prefer to do something in groups rather than on my own. It's easier. Much better. It's just getting the motivation to actually [be active].   So obviously you said as well, the motivation was really good at first but it didn't last very long, would you have felt comfortable to contact us?   - After the first year it was just like I probably don't want to be bothered so I just left it, and then it got to about September November time last year and [my camp friend] was like you never use it, and it was just like I never really see the point because I never know if they're going to reply and things. And well you’re a past camper; you've washed your hands of me. | Pattern of WLM: increased difficulty over time  Environmental triggers interfering with WLM behaviours  Fluctuating weight throughout the year  Had the knowledge  Self-efficacy  Forgetting WLM behaviours  Benefits of access to WLM resources  Dip in confidence biggest barrier to WLM  Recognising lifestyle sessions targeting psychosocial skills as beneficial  Social support of staff and peers  Had the knowledge-base  Wished for psychosocial skills to implement behaviours, supported by knowledge and resources  Drop in motivation  Psychosocial skills- drop in self-efficacy alongside weight regain  Abrupt end to WLM behaviours alongside termination of [intervention staff] support  Family support available  Not taking support offered from parents/ guardians  Dichotomous thinking patterns: sleep in equals wasted day  Psychosocial skills: self-efficacy to maintain lost weight  Environmental triggers  Perceived psychological effort to implement WLM behaviours  Psychologically  Conscious he should be more active  Perceived psychological effort to implement WLM behaviours  Avoided support when not prioritising WLM  Don’t expect to receive support from intervention staff  Loneliness- Perceived independence of WLM support |
| --- | --- |

**Steps 3-6: Developing themes, searching for connections, identifying patterns within and across themes**

| **Superordinate Theme** | **Themes, patterns & Connections** | **Supporting evidence: quotes** |
| --- | --- | --- |
| Theme 1: Behavioural Control and the Psychosocial Skills to Self-Regulate Weight Loss Maintenance | Intentions to maintain weight loss | *“Easy (pause) to begin with, and then it gets harder” (participant 1, male)*  “Yeah, quite prepared to begin with and then obviously over time… you begin to forget it.” (Participant 1, male)  *“I found it a lot easier at first because everything was quite fresh in my mind and it was like wanting to put everything into place but I’d say like after a few months I found I was finding it hard to stick to everything.” (Participant 6, female)* |
|  | Short-term high levels of self-efficacy (performance accomplishments, verbal persuasion) | “I was into my football, so I wanted to always be doing that.” (Participant 6, female)  “I used to have a cheeky little grin walking by, and it was just everyone saying well done.” (Participant 3, male)  *“my mum was like “oh you're so slim” and that would encourage me to do more stuff” (Participant 2, male)*  *“I used to give in [to temptations], but now I’m determined not to give in.” (Participant 5, female)*  *“I was quite motivated as soon as I came back so, yeah I think I was so much confident when I came back and I was motivated” (Participant 6, female)*  *“For me I was determined I had self-control I had motivation so without that [I’d had regained the weight]” (participant 4, female)* |
|  | Drop in self-efficacy following termination of formalised WLM intervention  (Linked to social support- impact on WLM behaviours without formal support pathway) | “Because the follow-on care had stopped, it was just like I didn't, I never really had a purpose to carry on with stuff like that.” (Participant 3, male)  “Especially when [intensive weight loss intervention staff] stopped the [calls], it just went bang” (participant 1, male)  *“Yeah it was really, really hard, you slip into old habits” (Participant 2, male)* |
|  | Lack of behavioural control  (Linked to social support- avoidance support from others) | *“It's just getting the motivation to actually [be active].” (participant 1, male)*  *“I put some weight back on and I didn't want to like, to be showing it's going backwards so I stopped [self-monitoring]” (Participant 4, female)*  *“It's hard to get out of the habit but then that's when you need control, self-control, things like that. And when I fell off track that's when I was going back to my old habits” (Participants 4, female)* |
| Theme 2: Delivering Effective Social Support | Continuity of Care (Relational continuity from intervention staff) | “I think it was just nice to hear like a friendly voice. It someone you know, that you've spent the summer with, you've got to know them they've got to know you” (participant 3, male)  *“Going like the look on their faces when they saw me lose weight each week like when we got on the weighing scales. That really pushed me on to keep going” (Participant 4, female)*  *“[I didn’t struggle to maintain weight loss] because once I finished my [residential intervention] I went straight back to [the community intervention]. Yeah, they helped a lot” (Participant 5, female)* |
|  | Familial support | “[my guardian has] always been supportive and he's always been there for me … he's been saying, we need to get something done you know your motivation … but I haven’t been listening to them” (Participant 1, male)  *“Mum offers me breakfast like porridge or poached eggs… and even my dad, he looks at labels more because he’s been to [the community intervention] with me and he tells me what I can and can’t have” (Participant 5, female)* |
|  | Letting staff down | *“If I come back [to the intervention] and I'm a lot lot bigger than I was then I thought some of the staff members [would] still be here and they’d be unhappy … because they put all that effort in it's just wasted a bit.” (Participant 2, male)*  *“My sister didn't want [to speak to intervention staff], because she went back to putting the weight back on” (Participant 4, female)* |
| Theme 3: Conflicting priorities and environmental triggers |  | “During the exam season, I didn’t [play football]” (participant 5, female)  “And then it's like, oooo, Halloween… then it gets to Christmas, and you're like ah, and then you just overeat, and it doesn't go off, and then it's my birthday nine days afterwards (laughs)” (participant 1, male)  “Obviously then I thought I was really busy, and then I look at it now, and I think Jesus I had absolutely nothing to do. All my priorities were in the wrong place.” (Participant 3, male)  *“Because of exams coming up. I stopped football” (Participant 6, female)* |
