# Supplementary material for: The lived experience of weight loss maintenance in young people
Source: Health Expect. 2023 Dec 28;27(1):e13955. doi: 10.1111/hex.13955 (PMC10768871; doi:10.1111/hex.13955)
Supplement: Supplementary file 2 — Supporting information. [file HEX-27-e13955-s001.docx]

Thank you for allowing me to visit you last week and see how your child was coming along. As you can imagine, throughout my visits I came across a number of experiences of weight change following camp. I now want to find out a bit more about those, both from the perspective of the child and you as a parent. I would be very grateful if I could arrange a phone call with you and your child. These will last approximately 30 minutes, however may be longer if you have lots to say!

| First of all I want to go back to when you first singed up your child to camp. As a family, were you happy about joining? |
| --- |
|  |
| Do you think your child changed at all as a result of camp? |
|  |
| Once home from camp was your child at all motivated or focussed to carry on losing weight? |
|  |
| What did you feel your role was in your child’s weight loss once they finished camp and came home? |
|  |
| Did you feel prepared to help your child? |
|  |
| Did your child teach you what they had learnt on camp? |
|  |

| What do you think your child struggled with most since camp? |
| --- |
|  |
| Is there anything you, as a parent/ guardian, have struggled with? |
|  |
| Could MoreLife have done anything differently to help you after camp? |
|  |
| What advice would you have liked before camp? |
|  |
| What advice would you have liked on camp? |
|  |
| What advice would you have liked after camp? |
|  |
| What advice would you give to future parents to help them support their child after camp? |
|  |

Next I would like to discuss some behaviours both campers and their families have identified as causing a struggle in their weight loss following camp. How has your child dealt with these?

| Fitting in activity around other commitments e.g. school, homework |
| --- |
|  |
| Snacking (what they are snacking on, how much they are snacking, when they are snacking?) |
|  |
| Secret eating |
|  |
| Motivation |
|  |
| What your child is eating away from home (e.g. at school, at friends houses, at parties) |
|  |
| Your child lying to you about what they have been doing/ eating |
|  |
| Your child lying to MoreLife about their progress during the phonecalls and visits |
|  |
| Support from friends/ family members |
|  |

Supplementary material A- Interview Guide
